# How Does Rescuer's Position Setting Impact Quality of Chest Compression: A Randomized Crossover Simulation Study on Unexperienced Clinicians

**DOI:** 10.1155/2024/9950885

**Published:** 2024-08-07

**Authors:** Nan Zhang, Jiangshan Wang, Yan Li, Jihai Liu, Huadong Zhu

**Affiliations:** Emergency Department The State Key Laboratory for Complex Severe and Rare Diseases Peking Union Medical College Hospital Chinese Academy of Medical Science and Peking Union Medical College, Beijing, China

## Abstract

**Background:**

High-quality chest compression (CC) is the crux of survival for cardiac arrest patients. While, rescuers' position setting relative to patients during CC was unrecommended in the present guidelines. We aimed to assess the impact of position settings on CC quality during cardiopulmonary resuscitation (CPR) and to test the heterogeneity related to rescuers' characteristics.

**Methods:**

We conducted randomized, crossover, simulation trials with clinical students unfamiliar with CPR. The participants received standard training on performing CC and were divided randomly into two groups. The two groups separately performed CC with standing and kneeling positions in turn, forming the crossover design. The trials were performed with standard manikin models. CC quality indicator data were recorded by the tracking and feedback system automatically.

**Result:**

156 participants finished at least one round of trial, with 126 participants finishing both rounds. Records for CC with kneeling and standing positions showed statistically significant differences in the correct rate, pause happening, average depth, and happening of over-depth compression. Regression analysis also implied that larger compression depths with the standing position were related to larger height and BMI of the participants.

**Conclusion:**

When performing CC, the standing position will lead to lower CC quality by larger chance of pause happening and over-depth compression. In addition, compression depth gaps between CC with kneeling and standing position were related with rescuer characteristics including height and BMI, with a threshold effect.

## 1. Introduction

The incidence and mortality of cardiac arrest are still very common [1, 2]. Clinical evidence and the guidelines hold that chest compression (CC) is the crux in series of cardiopulmonary resuscitation (CPR) operations. High-quality CC is associated with higher survival rate for patients with cardiac arrest [3–5]. CC quality can be measured by a system of indicators including the compression depth, rate, pause happening, chest recoil, and compression fraction [6]. Current guidelines recommend that a proper compression rate (100–120 times per minute), compression depth (5–6 centimeters), and lowest incidence of compression pause are basic components of the successful CPR [6, 7].

In clinical practice, performing high-quality CC by hands is still a major challenge for rescuers. Tiredness is the enemy of proper CC [8]. As a result, the guidelines suggested that the rescuers should be replaced in every two minutes [6, 7]. Rescuers' tiredness will differ according to their gender, age, and body mass index (BMI) [9]. While some factors exogenous to the rescuers can also influence the tiredness happening and affect CC quality [10]. Different CPR methods and varied relative position settings between the rescuer and patient are also key influencers [11, 12].

As for position settings, out-of-hospital patients with cardiac arrest will always receive CC on the ground, while in-hospital cardiac-arrest patients will more likely be chest compressed on fixed bed. Studies have shown that rescuer's relative position to the patient can influence the effectiveness of CC during CPR [13]. But the current guidelines from American Heart Association (AHA) [4] and European Resuscitation Council (ERC) [6] offer no clear recommendation for the rescuer's position settings.

There are great theoretical and practical values to understand deeper on proper position settings for CC during CPR. Consequently, we in this article tried to explore how and why the position settings of rescuers performing CC can impact the quality of CC with cardiac arrest manikin simulation.

## 2. Materials and Methods

### 2.1. Study Setting and Ethics Approval

This study was a single-center, randomized and crossover, observational simulation study. The research has been approved by the Ethics Review Committee of Beijing Union Medical College Hospital (with approval number: I-22PJ814). Before enrolling into the study, each participant had read and signed the informed consents. The research was carried out in accordance with requirements stated in the Helsinki Declaration of 1964 which was revised in 2013 [14].

### 2.2. Study Population

We included clinical students on their third or fourth year during the program of medicine doctor for eight years with basic medical training but were new to CPR operation. They were enrolled by the whole classes, with good representativeness of young clinicians in China. Before the formal trials, all participants received training on basic life support (BLS) according to the guidelines of American Heart Association (AHA), including being offered learning materials on CPR, lectures for 15 minutes on how to perform qualified chest compression, and standard practices on manikin models for five rounds with feedback about compression quality, followed by the BLS tests. When they had passed the tests, they were qualified to participate into the formal trials [15].

### 2.3. Detailed Design

We divided the qualified participants into two groups randomly. With random drawing lots, those with odd numbers were allocated into the first group and those with even numbers into the second group. In the first round of the trial, participants in the first group knelt beside the manikin on the ground (kneeling position setting) to perform CC, and participants in the second group stood beside the manikin which was on the bed of 60 centimeters high (standing position setting) when performing CC. The CC lasted for 2 minutes as the guidelines recommended. The participants in both groups were required to perform CC with the rate of 100–120 times per minute, the compression depth between 5 and 6 centimeters, and least pausing during CC, also as the guidelines recommended.

Then, both groups of participants rested for 60 minutes after the first round of trials. In the second round of trials, the first group performed CC with the standing position and the second group with the kneeling position. The trials at the second round were performed with the same time period and the same requirements as at the first round. In this way, the random crossover design was realized with the same participant performing CC with both standing and kneeling positions in turn.

During the two rounds of trials, no feedback information on compression quality were offered to the participants, letting them to fully simulate the practice by unexperienced rescuers. All the data on CC quality for trials in each round of every participant were automatically recorded by computer system linked to the manikins.

### 2.4. Outcome

The primary outcomes of the study were 8 kinds of CC quality indicators during CPR process, which will be compared between kneeling and standing position settings. The secondary outcome was the relation between the rescuer's height or BMI and CC quality differences with varied position settings.

### 2.5. Statistical Analysis

In this article, categorical variables were presented with frequencies in percentage. Also, continuous variables were presented as the mean value with standard deviation (S.D.) if the variables conform to normal distribution. Otherwise, they were presented using the median with interquartile range (IQR). The Shapiro–Wilk test was used to test whether the continuous variables conform to normal distribution. Categorical variables were compared between the records of CC performed with kneeling and standing positions using the Pearson Chi-squared test or Fisher's exact test. Besides, we use Student's *t*-test for data with normal distribution and the Mann–Whitney *U* test for variables with non-normal distribution. Regression analyses including threshold regression were systematically utilized [16].

The major statistical analyses and the best-fit line diagram of rescuer's height or BMI and CC depth gap were performed by STATA software (version 15.0). Bilateral *P* value less than 0.05 stood for statistically significant result.

## 3. Results

### 3.1. Study Participants

**Figure 1:**
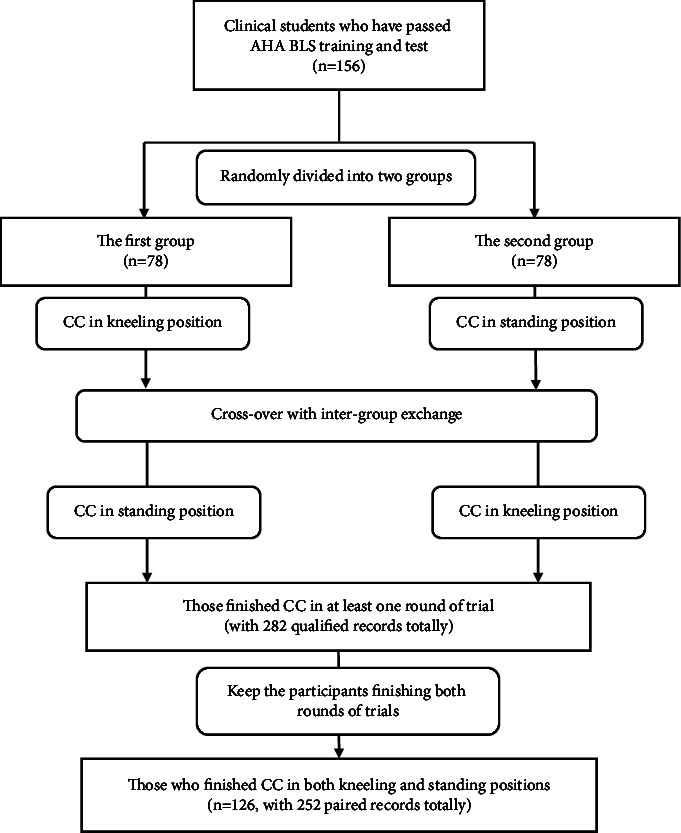
Trial flowchart of the participants' keeping.

Initially, the study enrolled 156 clinical students and gave them BLS training according to AHA guidelines. Those participants were divided into two groups randomly with 78 in each group. After the two rounds of crossover trials of CC with different positions, 282 qualified records were collected. While only 126 participants finished the both rounds of trials with 252 paired records. The detailed flowchart of participants' keeping is in Figure 1.

In Table 1, we provided the basic characteristics of the participants. Based on Panel A, the average age was around 22, and 61 of them were male (29.1%). The average height was 168.12 centimeters with the standard deviation (S.D.) of 7.63 centimeters, and the average weight was 60.85 kilograms with S.D. of 10.74 kilograms. Based on the height and weight of the participants, we obtained the BMI. The average BMI was 21.42, with S.D. of 2.72. For the gender heterogeneity, both male and female participants share the similar average age because we enrolled them with whole classes as the units. Males were taller and larger in weight than females, which showed significant difference (*P*-value <0.001). In addition, the males and females differ significantly on BMI as well. Besides, as for 126 participants finishing both rounds of the trials posted in Panel B, the statistic results of the participants' characteristics were similar.

### 3.2. The Quality of Chest Compressions

We compared CC quality between different position settings in Table 2. We found that CC with the standing position had a lower correct rate but more probability of pause happening and was about 2.48 millimeters deeper compared with the kneeling position (*P* value = 0.019). CC with the standing position had a larger over-depth compression happening rate and a lower less-depth compression happening rate (*P* value <0.001). While CC with varied position settings turned out to be similar on compression rate and similar chance of over-speed and less-speed compression. Besides, we kept the participants finishing both rounds of trials shown in Table A1 of Supplementary Materials, and the results stayed robust.

**Figure 2:**
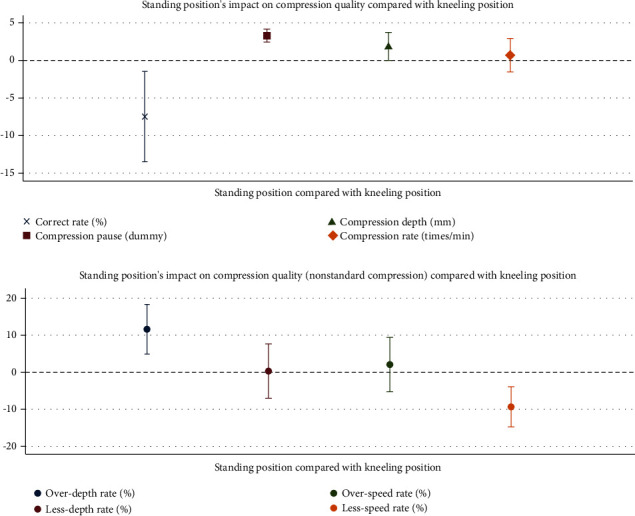
Regression results of standing position's impact on CC quality compared with the kneeling position.

Furthermore, we went on with the regression results for standing position's impact on CC quality compared with the kneeling position, with participants' characteristics including age, height, and BMI controlled. In Figure 2, the coefficients (varied dot shapes for varied dependent variables) and confidential interval (bars with 2 endpoints) were offered. The results showed a similar result with Table 2.

Then, we went on to analyze whether there was heterogeneity between male and female. Based on Table 3, males had larger chances of correct compression but also deeper compression, as well as a larger over-depth compression rate. Compression depth of females with kneeling position was more likely below the minimum standard of correct CC of 5 centimeters, which might cause a lower correct rate. The similar situation can be found when we kept 126 participants finishing both rounds of trials, as shown in Table A2 in Supplementary Materials.

**Figure 3:**
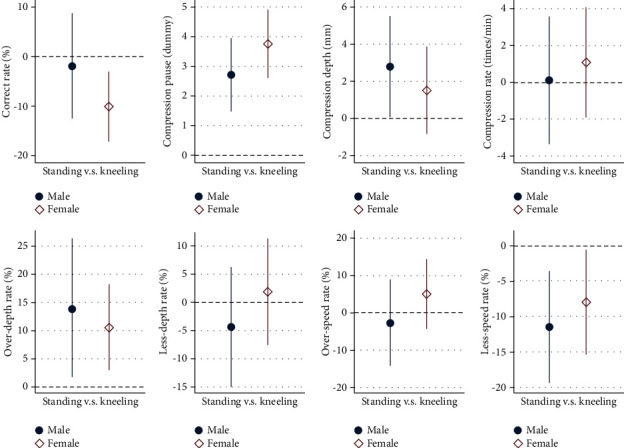
Regression results of standing position's heterogeneous impacts on CC quality of male and female participants compared with the kneeling position. *Note*. Coefficients (varied dot shapes for varied dependent variables) and confidential interval (bars with 2 endpoints) were marked for regression analysis with varied CC quality indicators as dependent variables.

In addition, we performed regression analysis of standing position setting's impact on CC quality compared with kneeling position setting, with personal characteristics controlled (Figure 3). For gender heterogeneity, standing position setting could lead to a lower correct rate in females but larger compression depth in male. We anticipated that the heterogeneities between male and female were due to the variety on personal characteristics such as height and BMI.

### 3.3. The Impact of Rescuer's Height and Weight on CC Quality

In this part, we provided analysis to explore the height and BMI of rescuers' impact on CC quality differences related to varied position settings, and especially whether threshold effect existed. The analysis unit was every individual record.

We choose the correct rate and compression depth as dependent variables because male and female varied significantly in those two CC quality indicators based on previous analyses. The threshold regression results are reported in Table 4. On the upper panel, we separately tested whether the standing position compared with the kneeling position would have different impacts on the correct rate for varied height and BMI ranges. Although there were some differences, the threshold effects were not significant based on Bootstrap *P* values. On the lower panel, the dependent variables were compression depth. Based on Bootstrap *P* values, the threshold effect existed. With larger height over 165 cm, the participants tended to have larger compression depth with the standing position compared to the kneeling position. Also, participants with larger BMI over 20.32 would show larger compression depth with the standing position. So, the differences between male and female might be due to the different rescuers' characteristics of height and BMI.

**Figure 4:**
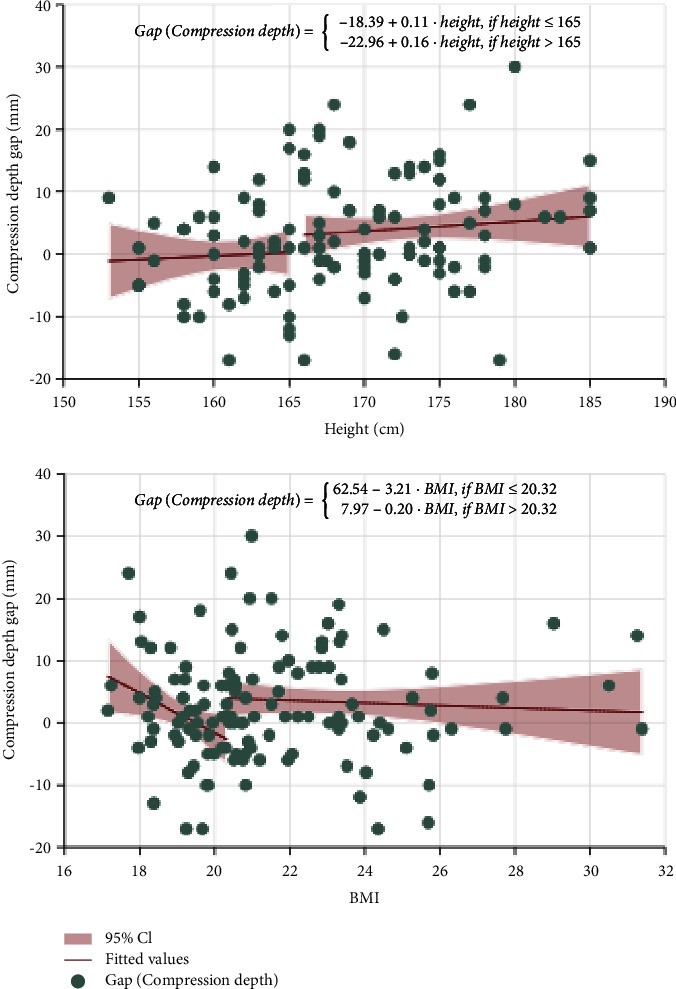
Relationship between rescuer's height/BMI and compression depth gap considering the threshold effect.

Enlightened by the findings, we went on to test the participant height and BMI's impact on the gaps of compression depth with standing and with the kneeling positions by the same rescuer. Thanks to the crossover design, we could gather data on compression depth with kneeling and standing positions for the same rescuer. Subtracting the average compression depth with the kneeling position from the average compression depth with standing position for the same rescuer, we had the gap of compression depth. In the upper part of Figure 4, we offered the best-fit line with rescuer's height as predictor for the gap of compression depth setting 165 cm as the threshold and found that one centimeter of height could lead to larger gaps of compression depth when the height of the rescuer was larger than 165 cm. In lower part of Figure 4, we analyzed the BMI as the threshold variable. One unit rise in BMI could cause the gap of compression depth by −3.21 if the BMI was smaller than 20.32 but causes little change if the BMI was larger than the threshold.

## 4. Discussion

For position setting in CC during CPR, rescuers' hand position [17], compression position on patients' body [18], and rescuers' relative position of standing or kneeling are all vital for CC quality. While rescuers' standing or kneeling choice is less explored under the latest guidelines and is our major focus. CC quality indices, like compression rate and depth, might always catch researchers' interests [17, 19]. While, impact of over-depth compression was always neglected, and was the interest of our study. Besides, studies on CC position setting were carried out with varied methods based on real-world data [18] or simulation design [20], applicable to different research situations. Among those methods, simulation trials on manikins providing steady trial environment and avoiding potential ethical problems was adopted in this study.

We included clinical students getting BLS trained for the first time, offering a homogenous and large-size participants sample. The design excluded potential interferences caused by experienced rescuers who can intentionally adjust their compression operation [21]. By simulating CC on manikins and comparing CC quality between kneeling and standing positions, we found that the standing position was more related to the deviating compression correctness, especially higher chance of pause happening and improper compression depth. The findings are important for clinical practice considering the potential harm that improper compression might cause [22, 23].

Previous studies implied that the compression depth is smaller when performed with the patients on bed compare to on ground with the kneeling position [24] but neglected the influence of rescuers individual characteristics. In this study, regression analyses showed that the compression depth difference between kneeling and standing positions was partly related to the rescuer's variety on height and BMI. The taller the rescuer was, the larger the compression depth would happen with the standing position, showing a clear threshold effect. While for BMI, with larger BMI over 20.32, the gap between compression depth with standing and kneeling positions would be steady.

Besides, previous studies were performed under the direction of AHA CPR guidelines of 2010 which did not set the maximum value for compression depth [25, 26]. Some researchers have mentioned that the proper compression depth can vary according to the age ranges of the patients [27] and pointed out the potential harm of over-depth compression. Our study was based on the latest guidelines [6, 7] and found that one major quality problem of CC was over-depth compression less explored.

Furthermore, we offered analysis on CC position setting choice during CPR in the simulation of practical situation, hopping to provide more enlightenment for clinical practice. Cho et al. studied how the height of bed can influence the outcomes of CC [28], while we did not adjust the bed height according to the rescuer's height because the settled bed height was more approximate to the real-world clinical practice. The bed adjustment process can cause delay for in-time CPR. As a result, to explore the best choice for rescuer's position setting based on rescuer's individual characteristics such as height and BMI can be more valuable to offer direct instruction for clinical practice.

Although we were confident that our study can offer some enlightenment, there were also limitations as well. First, this study was done with manikins instead of real patients considering the ethical reasons. In the real clinical situation, the position setting should be decided based on the actual environment, and the patients can vary greatly from each other, challenging the robustness of our findings. Second, the participants in our trials all performed CC in 2 minutes for both the kneeling and standing position settings, which is widely adopted with simulation trial of CC [20]. While in clinical practice, the CC period can be longer and tiredness in those two position settings can be different, which was not fully considered yet. Third, we enrolled the participants among clinical students instead of the experienced doctors and nurses. Even if the design can offer more pure treatment related to position settings only, the strategy utilized can be different from clinical practice where the experience of emergency rescuers might influence the CC quality [29]. Also, some latest studies found that the proper CC depth depends on the patient characteristics like external anteroposterior diameter [30]. CC depth can also be related to rescuer's handedness which is widely studied recently [19, 31]. As a result, whether the standing or kneeling position are more adoptable when considering patient individual characteristics and rescuer handedness calls for more explorations in the future.

## 5. Conclusions

When unexperienced rescuers perform CC during CPR, the position setting can influence CC quality. CC with the standing position beside patient on bed was related to larger probability of pause happening (53.85%) and higher over-depth compression rate (29.51%). The difference with varied position settings was related to rescuer's characteristics. With higher rescuer height, especially when the rescuers' height was larger than the threshold value of 165 cm, there was a bigger chance of over-depth compression for standing position setting. Consequently, the kneeling position, which can overcome the disturbance of rescuers' height, is more suggested for novice rescuers. All in all, for unexperienced rescuers performing CC, both proper position setting choice and proper training are important.

## Figures and Tables

**Table 1:** Baseline characteristics of the participants.

Panel A: original sample	Participants (*n* = 156)	Male (*n* = 61)	Female (*n* = 95)	*P*-value
Age, mean ± S.D. (years)	22.17 ± 2.42	22.21 ± 2.38	22.15 ± 2.45	0.869
Height, mean ± S.D. (cm)	168.12 ± 7.63	174.92 ± 5.22	163.76 ± 5.44	<0.001
Weight, mean ± S.D. (kg)	60.85 ± 10.74	69.76 ± 9.09	55.13 ± 7.27	<0.001
BMI, mean ± S.D. (kg/m^2^)	21.42 ± 2.72	22.80 ± 2.82	20.53 ± 2.26	<0.001

Panel B: final sample	Participants (*n* = 126)	Male (*n* = 47)	Female (*n* = 79)	*P*-value

Age, mean ± S.D. (years)	21.79 ± 2.43	21.66 ± 2.36	21.87 ± 2.48	0.635
Height, mean ± S.D. (cm)	168.46 ± 7.33	175.23 ± 4.87	164.43 ± 5.29	<0.001
Weight, mean ± S.D. (kg)	61.41 ± 10.91	70.82 ± 9.27	55.81 ± 7.41	<0.001
BMI, mean ± S.D. (kg/m^2^)	21.52 ± 2.80	23.06 ± 2.86	20.61 ± 2.34	<0.001

*Note*. All values were estimated with means (and with S.D. followed after “±”). *P* values were offered to tell the differences between the male and female participants.

**Table 2:** Evaluation of CC quality for kneeling and standing position settings.

	Position settings during CC		
	Kneeling (*n* = 152)	Standing (*n* = 130)	*P*-value
Correct rate (%)	30.60 ± 27.42	24.73 ± 25.32	0.068
Pause happening (%)	11.18 ± 31.62	53.85 ± 50.04	<0.001
Depth (mm)	50.70 ± 7.12	53.18 ± 10.48	0.019
Frequency (times/min)	113.28 ± 10.44	114.09 ± 8.63	0.483
Over-depth rate (%)	16.89 ± 24.96	29.51 ± 35.03	<0.001
Less-depth rate (%)	39.01 ± 34.56	37.04 ± 36.10	0.641
Over-speed rate (%)	22.76 ± 30.30	23.96 ± 31.96	0.748
Less-speed rate (%)	18.02 ± 30.37	6.55 ± 17.86	<0.001

*Note*. All values were estimated with means (and with S.D. followed after “±”). *P*-values were offered to tell the differences between the kneeling and standing position settings.

**Table 3:** Heterogeneity CC quality between male and female.

	Male			Female		
	Kneeling (*n* = 60)	Standing (*n* = 48)	*P*-value	Kneeling (*n* = 92)	Standing (*n* = 82)	*P*-value
Correct rate (%)	33.92 ± 28.35	31.33 ± 27.07	0.632	28.43 ± 26.72	20.87 ± 23.55	0.051
Pause happening (%)	13.33 ± 34.28	52.08 ± 50.48	<0.001	9.78 ± 29.87	54.88 ± 50.07	<0.001
Depth (mm)	54.00 ± 6.05	56.98 ± 7.85	0.028	44.55 ± 6.97	50.96 ± 11.20	0.087
Frequency (times/min)	114.32 ± 10.07	114.60 ± 6.98	0.867	112.61 ± 10.68	113.79 ± 9.49	0.443
Over-depth rate (%)	26.11 ± 28.15	40.26 ± 35.84	0.024	10.87 ± 20.66	23.21 ± 33.18	0.003
Less-depth rate (%)	23.67 ± 28.01	19.02 ± 2535	0.374	49.01 ± 34.89	47.59 ± 37.38	0.795
Over-speed rate (%)	24.77 ± 32.06	22.22 ± 30.61	0.677	21.45 ± 29.20	24.97 ± 32.86	0.456
Less-speed rate (%)	15.84 ± 28.08	1.91 ± 5.37	0.001	19.45 ± 31.85	9.26 ± 21.70	0.016

*Note*. All values were estimated with means (and with S.D. followed after “±”). *P* values were offered to tell the differences among varied subsamples.

**Table 4:** Threshold regression with varied height and BMI ranges.

	Coefficient	95% CI
Standing vs. kneeling position's impact on the correct rate		
Height ≤ 167.5	−7.53	(−15.70, 4.32)
Height > 167.5	−4.92	(−22.81, 9.32)
Bootstrap *P* value	0.146 (with no threshold effect)	
BMI ≤ 29.03	−5.7	(−34.40, 8.56)
BMI > 29.03	−14.95	(−38.43, 17.39)
Bootstrap *P* value	0.918 (with no threshold effect)	
Standing vs. kneeling position's impact on the compression depth		
Height ≤ 165	−0.61	(−3.46, 2.99)
Height > 165	3.99	(0.92, 6.43)
Bootstrap *P* value	0.017 (with threshold effect)	
BMI ≤ 20.32	1.02	(−2.17, 4.54)
BMI > 20.32	3.45	(0.58, 6.51)
Bootstrap *P* value	<0.001 (with threshold effect)	

*Note*. Coefficients of regressions for different models were offered with 95% confidence interval (CI) in the parentheses followed. Bootstrap *P* values were offered to tell whether the threshold effect existed.

## Data Availability

The datasets used and analyzed during the current study are available from the corresponding author upon reasonable request.

## Supplementary Materials

Supplementary Materials Additional tables which can support the understanding of the paper are provided in the Supplementary Materials. Table A1 of Supplementary Materials offers evaluation of CC quality for kneeling and standing position settings with participants finishing both rounds of trials. Table A2 of Supplementary Materials offers heterogeneous CC quality of male and female participants finishing both rounds of trials.

## References

[1] Yan S., Gan Y., Jiang N. (2020). The global survival rate among adult out-of-hospital cardiac arrest patients who received cardiopulmonary resuscitation: a systematic review and meta-analysis. *Critical Care*.

[2] Holmberg J., Granfeldt A., Stankovic N., Andersen L. (2022). Intra-cardiac arrest transport and survival from out-of-hospital cardiac arrest: a nationwide observational study. *Resuscitation*.

[3] Rea T. D., Fahrenbruch C., Culley L. (2010). CPR with chest compression alone or with rescue breathing. *New England Journal of Medicine*.

[4] Olasveengen T. M., Mancini M. E., Perkins G. D. (2020). Adult basic life support: international consensus on cardiopulmonary resuscitation and emergency cardiovascular care science with treatment recommendations. *Resuscitation*.

[5] Talikowska M., Tohira H., Finn J. (2015). Cardiopulmonary resuscitation quality and patient survival outcome in cardiac arrest: a systematic review and meta-analysis. *Resuscitation*.

[6] Olasveengen T. M., Semeraro F., Ristagno G. (2021). European resuscitation Council guidelines 2021: basic life support. *Resuscitation*.

[7] Panchal A. R., Bartos J. A., Cabañas J. G. (2020). Part 3: adult basic and advanced life support: 2020 American Heart association guidelines for cardiopulmonary resuscitation and emergency cardiovascular care. *Circulation*.

[8] Wik L., Kramer-Johansen J., Myklebust H. (2005). Quality of cardiopulmonary resuscitation during out-of-hospital cardiac arrest. *JAMA*.

[9] Hong D. Y., Park S. O., Lee K. R., Baek K. J., Shin D. H. (2012). A different rescuer changing strategy between 30:2 cardiopulmonary resuscitation and hands-only cardiopulmonary resuscitation that considers rescuer factors: a randomised cross-over simulation study with a time-dependent analysis. *Resuscitation*.

[10] Jo C. H., Cho G. C., Ahn J. H., Park Y. S., Lee C. H. (2015). Rescuer-limited cardiopulmonary resuscitation as an alternative to 2-min switched CPR in the setting of inhospital cardiac arrest: a randomised cross-over study. *Emergency Medicine Journal*.

[11] Barcala-Furelos R., Abelairas-Gomez C., Romo-Perez V., Palacios-Aguilar J. (2013). Effect of physical fatigue on the quality CPR: a water rescue study of lifeguards: physical fatigue and quality CPR in a water rescue. *The American Journal of Emergency Medicine*.

[12] Foo N.-P., Chang J.-H., Lin H. J., Guo H. R. (2010). Rescuer fatigue and cardiopulmonary resuscitation positions: a randomized controlled crossover trial. *Resuscitation*.

[13] Hong C. K., Park S. O., Jeong H. H. (2014). The most effective rescuer’s position for cardiopulmonary resuscitation provided to patients on beds: a randomized, controlled, crossover mannequin study. *The Journal of Emergency Medicine*.

[14] World Medical Association (2013). World medical association declaration of Helsinki: ethical principles for medical research involving human subjects. *JAMA*.

[15] Kim S., You J. S., Lee H. S. (2013). Quality of chest compressions performed by inexperienced rescuers in simulated cardiac arrest associated with pregnancy. *Resuscitation*.

[16] Hou W. H., Chuang H. Y., Lee M. L. T. (2016). A threshold regression model to predict return to work after traumatic limb injury. *Injury*.

[17] O’Connell K. J., Sandler A., Dutta A. (2023). The effect of hand position on chest compression quality during CPR in young children: findings from the Videography in Pediatric Resuscitation (VIPER) collaborative. *Resuscitation*.

[18] Berruti A., McCann-Pineo M., Li T. (2023). Positioned for success: a novel exploration of changes to chest compressions during cardiopulmonary resuscitation and associated patient outcomes. *Annals of Emergency Medicine*.

[19] Kim J., Oh J. H., Min K., Kim D. H. (2024). Comparisons of the vertical one-handed chest compressions according to the rescuer’s handedness. *The American Journal of Emergency Medicine*.

[20] Marks S., Shaffer L., Zehnder D., Aeh D., Prall D. M. (2023). Under pressure: what individual characteristics lead to performance of high-quality chest compressions during CPR practice sessions?. *Resuscitation Plus*.

[21] Chi C. H., Tsou J. Y., Su F. C. (2008). Effects of rescuer position on the kinematics of cardiopulmonary resuscitation (CPR) and the force of delivered compressions. *Resuscitation*.

[22] Nagashima F., Inoue S., Oda T. (2024). The SAVE-J II study group. Optimal chest compression for cardiac arrest until the establishment of ECPR: secondary analysis of the SAVE-J II study. *The American Journal of Emergency Medicine*.

[23] Telec W., Kłosiewicz T., Zalewski R., Żukowska-Karolak J., Baszko A., Puślecki M. (2021). Impact of postshock transcutaneous pacing on chest compression quality during resuscitation: a simulation-based pilot study. *Emergency Medicine International*.

[24] Jäntti H., Silfvast T., Turpeinen A., Kiviniemi V., Uusaro A. (2009). Quality of cardiopulmonary resuscitation on manikins: on the floor and in the bed. *Acta Anaesthesiologica Scandinavica*.

[25] Idris A. H., Guffey D., Aufderheide T. P. (2012). Relationship between chest compression rates and outcomes from cardiac arrest. *Circulation*.

[26] Idris A. H., Guffey D., Pepe P. E. (2015). Chest compression rates and survival following out-of-hospital cardiac arrest. *Critical Care Medicine*.

[27] Lee J. H., Han S. K., Na J. U. (2019). Current guideline of chest compression depth for children of all ages may be too deep for younger children. *Emergency Medicine International*.

[28] Cho J., Oh J. H., Park Y. S., Park I. C., Chung S. P. (2009). Effects of bed height on the performance of chest compressions. *Emergency Medicine Journal*.

[29] De Vaux L. A., Cassella N., Sigovitch K. (2021). Resuscitation team roles and responsibilities: in-hospital cardiopulmonary arrest teams. *Critical Care Nursing Clinics*.

[30] Sanguanwit P., Saksobhavivat N., Phattharapornjaroen P. (2024). Appropriateness of recommended chest compression depths for cardiopulmonary resuscitation based on chest computed tomography parameters among Thai population: a multicenter retrospective study. *Resuscitation Plus*.

[31] Marquis A., Douillet D., Morin F. (2023). Comparison of chest compression quality between the overlap hands and interlocking hands techniques: a randomised cross-over trial. *The American Journal of Emergency Medicine*.

