# Supplementary material for: How Does Rescuer's Position Setting Impact Quality of Chest Compression: A Randomized Crossover Simulation Study on Unexperienced Clinicians
Source: Emerg Med Int. 2024 Aug 7;2024:9950885. doi: 10.1155/2024/9950885 (PMC11325014; doi:10.1155/2024/9950885)
Supplement: Supplementary Materials — Additional tables which can support the understanding of the paper are provided in the Supplementary Materials. Table A1 of Supplementary Materials offers evaluation of CC quality for kneeling and standing position settings with participants finishing both rounds of trials. Table A2 of Supplementary Materials offers heterogeneous CC quality of male and female participants finishing both rounds of trials. [file 9950885.f1.docx]

**Supplementary Materials**

Table A1. Evaluation of CC quality for kneeling and standing position settings with participants finishing both rounds of trials

|  | Position settings during CC | | |
| --- | --- | --- | --- |
|  | Kneeling (n=126) | Standing (n=126) | p-value |
| Correct rate (%) | 31.65±28.16 | 24.64±25.47 | 0.039 |
| Pause happening (%) | 8.73±28.34 | 55.56±46.76 | <0.001 |
| Depth (mm) | 50.50±7.04 | 53.10±10.44 | 0.022 |
| Frequency (times/min) | 112.75±9.68 | 114.01±8.75 | 0.282 |
| Over-deep rate (%) | 15.67±24.07 | 29.51±34.95 | <0.001 |
| Less-deep rate (%) | 40.74±34.81 | 37.19±36.48 | 0.430 |
| Over-speed rate (%) | 20.28±27.86 | 23.95±32.38 | 0.336 |
| Less-speed rate (%) | 10.46±23.17 | 6.72±18.11 | 0.155 |

Note: All values were estimated with means (and with S.D. followed after “±”). P-values were offered to tell the differences between the kneeling and standing position settings.

Table A2. Heterogeneous CC quality of male and female participants finishing both rounds of trials

|  | Male | | | Female | | |
| --- | --- | --- | --- | --- | --- | --- |
|  | Kneeling  (n=47) | Standing  (n=47) | p-value | Kneeling  (n=79) | Standing  (n=79) | p-value |
| Correct rate (%) | 35.88±29.40 | 31.52±27.33 | 0.458 | 29.12±27.27 | 20.54±23.53 | 0.036 |
| Pause happening (%) | 10.64±31.17 | 53.19±50.44 | <0.001 | 7.59±26.66 | 56.96±49.83 | <0.001 |
| Depth (mm) | 53.17±6.29 | 57.19±7.80 | 0.007 | 48.91±7.02 | 50.66±11.08 | 0.238 |
| Frequency (times/min) | 35.88±29.40 | 31.52±27.33 | 0.458 | 29.12±27.27 | 20.54±23.53 | 0.036 |
| Over-deep rate (%) | 23.09±26.81 | 41.10±35.75 | 0.007 | 11.26±21.27 | 22.63±32.80 | 0.011 |
| Less-deep rate (%) | 27.71±30.11 | 18.28±25.10 | 0.103 | 48.49±35.27 | 48.44±37.65 | 0.992 |
| Over-speed rate (%) | 20.63±28.32 | 22.03±30.92 | 0.819 | 20.08±27.76 | 25.09±33.36 | 0.306 |
| Less-speed rate (%) | 6.97±17.43 | 1.95±5.42 | 0.063 | 12.53±25.88 | 9.56±22.06 | 0.428 |

Note: All values were estimated with means (and with S.D. followed after “±”). P-values were offered to tell the differences between the kneeling and standing position settings.
